# Cynaratriol, a sesquiterpene lactone from *Centaurea musimomum*

**DOI:** 10.1107/S1600536809026701

**Published:** 2009-07-15

**Authors:** Matías López-Rodríguez, Victor P. García, Hanêne Zater, Samir Benayache, Fadila Benayache

**Affiliations:** a Instituto de Bioorgánica "A. González", Universidad de La Laguna, Astrofísico Francisco Sánchez, 2, 38206 La Laguna, Tenerife, Spain; b Instituto de Productos Naturales y Agrobiología del CSIC, Instituto de Bioorgánica "A. González", Universidad de La Laguna, Astrofísico Francisco Sánchez, 2, 38206 La Laguna, Tenerife, Spain; c Laboratoire de Phytochemie et Analyses Physico-Chimiques et Biologiques, Equipe Associée à l’ANDRS, Université Mentouri, Route de Aïn El Bey, 25000 Constantine, Algeria; d Laboratoire de Valorisation des Resources Naturelles et Synthèse de Substances, Bioactives, Equipe Associée à l’ANDRS, Université Mentouri, Route de Aïn El Bey, 25000 Constantine, Algeria

## Abstract

The title compound [systematic name: 3,8-dihydr­oxy-3-(hydroxy­meth­yl)-9-methyl-6-methyl­enedeca­hydro­azuleno[4,5-*b*]furan-2(3*H*)-one], C_15_H_22_O_5_, is a sesquiterpene lactone showing the typical tricyclic guaianolide skeleton which has been isolated, together with other related metabolites, from the plant *Centaurea musimomum*. The present study confirms the mol­ecular structure, assigned by ^1^H NMR and MS spectroscopy, as well as the the 11β-hydroxy­methyl, 3β-hydr­oxy and 4α-methyl stereochemistry. The crystal structure is built through a network of O—H⋯O hydrogen bonds involving the three hydroxyl groups that are present in the mol­ecular skeleton.

## Related literature

For the ethyl acetate soluble extract of *Centaurea musimomum*, an endemic specie from Algeria, see: Quezel & Santa (1963 ▶). For the structures of several guaianolide type sesquiterpene lactones isolated from the chloro­form-soluble part of *Centaurea musimomum*, see: Medjroubi *et al.* (1997 ▶, 2003 ▶, 2005 ▶); González-Platas *et al.* (1999 ▶). Cynaratriol was previously isolated from *Cynara* species, see: von Heinz *et al.* (1979 ▶). For related structures, see: Oksuz *et al.* (1993 ▶); González-Platas *et al.* (1999 ▶).
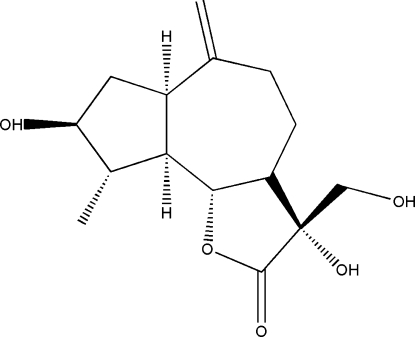


## Experimental

### 

#### Crystal data

C_15_H_22_O_5_
*M* *_r_* = 282.33
Orthorhombic, 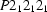

*a* = 8.417 (4) Å
*b* = 9.919 (3) Å
*c* = 17.187 (8) Å
*V* = 1434.9 (10) Å^3^
*Z* = 4
Mo *K*α radiation
μ = 0.10 mm^−1^
*T* = 293 K
0.40 × 0.30 × 0.25 mm

#### Data collection

Nonius KappaCCD diffractometer
Absorption correction: none
8829 measured reflections
2054 independent reflections
1847 reflections with *I* > 2σ(*I*)
*R*_int_ = 0.038

#### Refinement

*R*[*F* ^2^ > 2σ(*F* ^2^)] = 0.042
*wR*(*F* ^2^) = 0.101
*S* = 1.11
2054 reflections
194 parameters
H atoms treated by a mixture of independent and constrained refinement
Δρ_max_ = 0.21 e Å^−3^
Δρ_min_ = −0.17 e Å^−3^

### 

Data collection: *COLLECT* (Nonius, 2000 ▶); cell refinement: *SCALEPACK* (Otwinowski & Minor, 1997 ▶); data reduction: *SCALEPACK* and *DENZO* (Otwinowski & Minor, 1997 ▶); program(s) used to solve structure: *SIR97* (Altomare *et al.*, 1999 ▶); program(s) used to refine structure: *SHELXL97* (Sheldrick, 2008 ▶); molecular graphics: *PLATON* (Spek, 2009 ▶); software used to prepare material for publication: *WinGX* (Farrugia, 1999 ▶).

## Supplementary Material

Crystal structure: contains datablocks global, I. DOI: 10.1107/S1600536809026701/bx2222sup1.cif

Structure factors: contains datablocks I. DOI: 10.1107/S1600536809026701/bx2222Isup2.hkl

Additional supplementary materials:  crystallographic information; 3D view; checkCIF report

## Figures and Tables

**Table 1:** Hydrogen-bond geometry (Å, °)

*D*—H⋯*A*	*D*—H	H⋯*A*	*D*⋯*A*	*D*—H⋯*A*
O4—H4*O*⋯O1^i^	0.84 (3)	1.93 (3)	2.756 (2)	169 (3)
O5—H5*O*⋯O3^ii^	0.86 (3)	1.99 (3)	2.844 (2)	176 (3)
O1—H1*O*⋯O3^iii^	0.76 (4)	2.26 (4)	2.978 (3)	159 (4)

Symmetry codes: (i) 
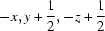
; (ii) 
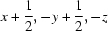
; (iii) 
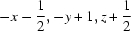
.

**Fig. 1.**
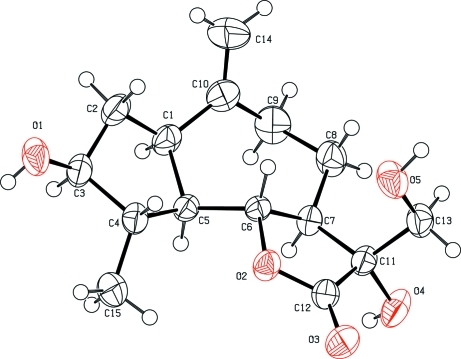
Molecular structure of cynaratriol represented showing displacement ellipsoids at the 50% probability level.

**Fig. 2.**
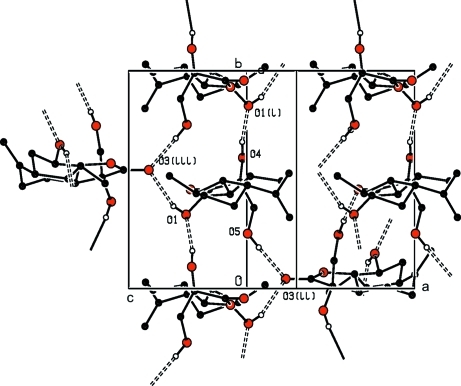
A view of the hydrogen-bonding network. Hydrogen atoms not involved in the O—H···O interactions have been omitted.

## supplementary crystallographic information

### Comment

The *Centaurea* genus plants are a natural source of various type of sesquiterpenic lactones, many of which have been shown to be biologically active: Medjroubi, Benayache & Bermejo. In connection with a systematic study of this genus, we have investigated the ethyl acetate soluble extract of *Centaurea musimomum*, an endemic specie from Algeria: Quezel & Santa, (1963). Our previous phytochemical study of the chloroform soluble part led to the isolation an molecular structure determination of several guaianolide type sesquiterpene lactones: Medjroubi *et al.*, 1997; González-Platas *et al.* 1999; Medjroubi *et al.*, 2003. Cynaratriol was previously isolated from *Cynara* species (Heinz, Thiele & Pretsch, 1979). In this work we report the isolation and the molecular and crystal structure determination of the title compound, which it is described for the first time as a metabolite for the *Centaurea* genus. The lack of suitable anomalous scatters did not allow us to reliably determine the absolute structure and that shown was chosen to be the same as that the, close related, 4β,15-Dihydro-3-dehydrosolstiatilin A (González-Platas *et al.*, 1999) and its acetate (Oksuz, Clark & Herz, 1993).

### Refinement

The H-atoms of the hydroxyl groups were located on difference-Fourier map and freely refined. All other H atoms were positioned with idealized geometry: C—H = 0.98(CH_3_), 0.99(CH_2_), 1.00(CH) Å and included in the refinement in a riding-model approximation with *U*_iso_(H) = 1.2*U*_eq_(C) and *U*_iso_(H) = 1.5*U*_eq_ for methyl. The lack of suitable anomalous scatters did not allow us to reliably determine the absolute structure according to the Flack parameters: -10 (10) and, therefore, the Friedel pairs were merged prior to the final refinement.

### Crystal data

**Table:**

C_15_H_22_O_5_	*F*(000) = 608
*M**_r_* = 282.33	*D*_x_ = 1.307 Mg m^−^^3^
Orthorhombic, *P*2_1_2_1_2_1_	Mo *K*α radiation, λ = 0.71073 Å
Hall symbol: P 2ac 2ab	Cell parameters from 7053 reflections
*a* = 8.417 (4) Å	θ = 3.2–28.6°
*b* = 9.919 (3) Å	µ = 0.10 mm^−^^1^
*c* = 17.187 (8) Å	*T* = 293 K
*V* = 1434.9 (10) Å^3^	Block, colourless
*Z* = 4	0.40 × 0.30 × 0.25 mm

### Data collection

**Table:**

Enraf–Nonius KappaCCD diffractometer	1847 reflections with *I* > 2σ(*I*)
Radiation source: fine-focus sealed tube	*R*_int_ = 0.038
graphite	θ_max_ = 28.6°, θ_min_ = 3.2°
Detector resolution: 9 pixels mm^-1^	*h* = −10→11
φ and ω scans	*k* = −12→12
8829 measured reflections	*l* = −22→23
2054 independent reflections	

### Refinement

**Table:**

Refinement on *F*^2^	0 restraints
Least-squares matrix: full	H atoms treated by a mixture of independent and constrained refinement
*R*[*F*^2^ > 2σ(*F*^2^)] = 0.042	*w* = 1/[σ^2^(*F*_o_^2^) + (0.0447*P*)^2^ + 0.2149*P*] where *P* = (*F*_o_^2^ + 2*F*_c_^2^)/3
*wR*(*F*^2^) = 0.101	(Δ/σ)_max_ = 0.01
*S* = 1.11	Δρ_max_ = 0.21 e Å^−^^3^
2054 reflections	Δρ_min_ = −0.17 e Å^−^^3^
194 parameters	

### Special details

**Table:**

Experimental. *Centaurea musimomum L*.was collected in June 2002 in Didouche Mourad (Constantine) Algeria and authenticated by Professor Nadra Khalfallah (Quezel & Santa, 1963). A voucher specimen was deposited in the herbarium of the Department of Nature and Life Sciences, Mentouri University, Constantine. Air-dried leaves (339 g) and air-dried flowers (202 g) were separately macerated, three times for 24 h, at room temperature with a mixture of methanol-water (70:30). The filtrates were concentrated and successsively extracted with petrol, chloroform, ethyl acetate and n-butanol. The ethyl acetate phases yield, after drying and solvent evaporation 10.8 g and 4.9 g respectively. Analysis by TLC on silica-gel plates showed no significant differences between the leaves and the flowers extract and they were mixed. A part (13 g) of the combined extract was chromatographed on a 230–400 mesh silica gel (325 g) column with chloroform-acetone mixtures of increasing polarity as elution solvents to yield the title compound: cynaratriol together with other related sesquiterpene lactones.The molecular formula C~15~ H~22~ O~5~ was deduced from its high resolution MS spectrum which shows a molecular ion at *m/z*=280.1329. The ^13Ĉ and ^1ĤNMR data are very similar to those of 4β,15-Dihydro-3-dehydrosolstiatilin A (Medjroubi *et al.*, 2003), the differences arises from the replacement of the oxo group at C3 by an hydroxyl group.
Geometry. All s.u.'s (except the s.u. in the dihedral angle between two l.s. planes) are estimated using the full covariance matrix. The cell s.u.'s are taken into account individually in the estimation of s.u.'s in distances, angles and torsion angles; correlations between s.u.'s in cell parameters are only used when they are defined by crystal symmetry. An approximate (isotropic) treatment of cell s.u.'s is used for estimating s.u.'s involving l.s. planes.
Refinement. Refinement of *F*^2^ against ALL reflections. The weighted *R*-factor *wR* and goodness of fit *S* are based on *F*^2^, conventional *R*-factors *R* are based on *F*, with *F* set to zero for negative *F*^2^. The threshold expression of *F*^2^ > 2σ(*F*^2^) is used only for calculating *R*-factors(gt) *etc*. and is not relevant to the choice of reflections for refinement. *R*-factors based on *F*^2^ are statistically about twice as large as those based on *F*, and *R*- factors based on ALL data will be even larger.

### Fractional atomic coordinates and isotropic or equivalent isotropic displacement parameters (Å^2^)

**Table:**

	*x*	*y*	*z*	*U*_iso_*/*U*_eq_	
O1	−0.0496 (3)	0.33998 (17)	0.44392 (9)	0.0577 (5)	
H1O	−0.107 (5)	0.379 (4)	0.469 (2)	0.108 (15)*	
O2	−0.14872 (16)	0.42543 (15)	0.15121 (8)	0.0379 (3)	
O3	−0.30157 (18)	0.45503 (19)	0.04747 (9)	0.0540 (4)	
O4	−0.0339 (3)	0.60068 (15)	−0.01439 (9)	0.0521 (5)	
H4O	−0.020 (4)	0.673 (3)	0.0102 (18)	0.070 (9)*	
O5	0.0257 (3)	0.25259 (15)	0.02916 (9)	0.0536 (5)	
H5O	0.074 (3)	0.190 (3)	0.0041 (15)	0.052 (7)*	
C1	0.2066 (2)	0.4743 (2)	0.28881 (12)	0.0388 (5)	
H1	0.2376	0.5613	0.3112	0.047*	
C2	0.1768 (3)	0.3797 (2)	0.35773 (12)	0.0472 (5)	
H2A	0.1727	0.2863	0.3412	0.057*	
H2B	0.258	0.3898	0.3973	0.057*	
C3	0.0174 (3)	0.4275 (2)	0.38653 (11)	0.0422 (5)	
H3	0.0292	0.5179	0.4088	0.051*	
C4	−0.0815 (2)	0.4369 (2)	0.31246 (11)	0.0358 (4)	
H4	−0.1096	0.3453	0.2962	0.043*	
C5	0.0359 (2)	0.49556 (18)	0.25231 (10)	0.0308 (4)	
H5	0.0163	0.5926	0.2478	0.037*	
C6	0.0215 (2)	0.43313 (19)	0.17180 (10)	0.0289 (4)	
H6	0.0675	0.3424	0.1721	0.035*	
C7	0.0949 (2)	0.51598 (19)	0.10596 (10)	0.0326 (4)	
H7	0.0813	0.6111	0.12	0.039*	
C8	0.2719 (3)	0.4939 (3)	0.09333 (13)	0.0476 (5)	
H8A	0.3076	0.5493	0.0502	0.057*	
H8B	0.2906	0.4003	0.0798	0.057*	
C9	0.3682 (3)	0.5293 (3)	0.16608 (14)	0.0542 (6)	
H9A	0.4804	0.5268	0.1533	0.065*	
H9B	0.3425	0.6206	0.1819	0.065*	
C10	0.3375 (2)	0.4353 (2)	0.23343 (13)	0.0461 (5)	
C11	−0.0147 (2)	0.48927 (19)	0.03617 (10)	0.0346 (4)	
C12	−0.1702 (2)	0.4552 (2)	0.07626 (11)	0.0373 (4)	
C13	0.0313 (3)	0.37152 (19)	−0.01605 (12)	0.0422 (5)	
H13A	−0.042	0.3648	−0.0594	0.051*	
H13B	0.1375	0.3848	−0.0366	0.051*	
C14	0.4258 (3)	0.3269 (3)	0.24326 (18)	0.0706 (8)	
H14A	0.4085	0.2709	0.2858	0.085*	
H14B	0.5055	0.3065	0.2077	0.085*	
C15	−0.2339 (3)	0.5172 (3)	0.32204 (14)	0.0529 (6)	
H15B	−0.2088	0.6069	0.3391	0.079*	
H15A	−0.3005	0.4742	0.36	0.079*	
H15C	−0.2887	0.5213	0.2731	0.079*	

### Atomic displacement parameters (Å^2^)

**Table:**

	*U*^11^	*U*^22^	*U*^33^	*U*^12^	*U*^13^	*U*^23^
O1	0.0988 (15)	0.0417 (8)	0.0325 (8)	0.0184 (9)	0.0167 (9)	0.0085 (6)
O2	0.0299 (6)	0.0527 (8)	0.0313 (7)	−0.0041 (6)	−0.0031 (6)	−0.0017 (6)
O3	0.0408 (8)	0.0734 (11)	0.0476 (9)	0.0025 (8)	−0.0155 (7)	−0.0084 (8)
O4	0.0908 (13)	0.0333 (7)	0.0323 (8)	−0.0003 (8)	−0.0162 (9)	0.0022 (6)
O5	0.0823 (13)	0.0323 (7)	0.0462 (9)	0.0082 (8)	0.0134 (9)	−0.0018 (7)
C1	0.0402 (10)	0.0410 (10)	0.0353 (10)	−0.0029 (8)	−0.0124 (8)	−0.0052 (8)
C2	0.0600 (13)	0.0485 (11)	0.0330 (11)	0.0082 (11)	−0.0148 (10)	−0.0014 (9)
C3	0.0679 (14)	0.0321 (9)	0.0268 (9)	0.0063 (10)	−0.0025 (10)	−0.0004 (7)
C4	0.0454 (10)	0.0334 (9)	0.0286 (9)	−0.0013 (9)	0.0015 (8)	0.0000 (7)
C5	0.0344 (9)	0.0299 (8)	0.0280 (8)	0.0005 (7)	−0.0039 (7)	−0.0003 (7)
C6	0.0264 (8)	0.0318 (8)	0.0286 (9)	0.0000 (7)	−0.0024 (7)	−0.0003 (7)
C7	0.0358 (9)	0.0345 (9)	0.0276 (9)	−0.0040 (8)	−0.0006 (8)	−0.0009 (7)
C8	0.0375 (11)	0.0649 (14)	0.0403 (11)	−0.0067 (11)	0.0058 (9)	−0.0016 (11)
C9	0.0317 (10)	0.0745 (16)	0.0563 (14)	−0.0137 (11)	0.0001 (10)	−0.0048 (12)
C10	0.0295 (9)	0.0611 (12)	0.0476 (12)	−0.0039 (10)	−0.0109 (9)	−0.0071 (10)
C11	0.0464 (10)	0.0300 (8)	0.0272 (9)	0.0007 (8)	−0.0045 (8)	−0.0004 (7)
C12	0.0395 (10)	0.0387 (10)	0.0338 (10)	0.0014 (9)	−0.0068 (8)	−0.0092 (8)
C13	0.0572 (13)	0.0373 (10)	0.0320 (10)	0.0021 (10)	0.0015 (10)	−0.0045 (8)
C14	0.0441 (13)	0.088 (2)	0.0794 (19)	0.0196 (15)	−0.0051 (14)	−0.0036 (16)
C15	0.0459 (12)	0.0723 (15)	0.0405 (12)	0.0069 (12)	0.0112 (10)	0.0035 (11)

### Geometric parameters (Å, °)

**Table:**

O1—C3	1.430 (3)	C5—H5	0.98
O1—H1O	0.76 (4)	C6—C7	1.529 (3)
O2—C12	1.334 (2)	C6—H6	0.98
O2—C6	1.477 (2)	C7—C8	1.522 (3)
O3—C12	1.211 (2)	C7—C11	1.536 (3)
O4—C11	1.415 (2)	C7—H7	0.98
O4—H4O	0.84 (3)	C8—C9	1.531 (3)
O5—C13	1.413 (3)	C8—H8A	0.97
O5—H5O	0.86 (3)	C8—H8B	0.97
C1—C10	1.507 (3)	C9—C10	1.509 (3)
C1—C2	1.531 (3)	C9—H9A	0.97
C1—C5	1.582 (3)	C9—H9B	0.97
C1—H1	0.98	C10—C14	1.317 (4)
C2—C3	1.507 (3)	C11—C12	1.517 (3)
C2—H2A	0.97	C11—C13	1.523 (3)
C2—H2B	0.97	C13—H13A	0.97
C3—C4	1.524 (3)	C13—H13B	0.97
C3—H3	0.98	C14—H14A	0.93
C4—C15	1.519 (3)	C14—H14B	0.93
C4—C5	1.544 (3)	C15—H15B	0.96
C4—H4	0.98	C15—H15A	0.96
C5—C6	1.521 (2)	C15—H15C	0.96
			
C3—O1—H1O	110 (3)	C8—C7—H7	106.7
C12—O2—C6	110.58 (15)	C6—C7—H7	106.7
C11—O4—H4O	110 (2)	C11—C7—H7	106.7
C13—O5—H5O	108.2 (17)	C7—C8—C9	111.64 (18)
C10—C1—C2	116.82 (18)	C7—C8—H8A	109.3
C10—C1—C5	116.63 (16)	C9—C8—H8A	109.3
C2—C1—C5	103.87 (16)	C7—C8—H8B	109.3
C10—C1—H1	106.2	C9—C8—H8B	109.3
C2—C1—H1	106.2	H8A—C8—H8B	108
C5—C1—H1	106.2	C10—C9—C8	113.23 (19)
C3—C2—C1	101.96 (17)	C10—C9—H9A	108.9
C3—C2—H2A	111.4	C8—C9—H9A	108.9
C1—C2—H2A	111.4	C10—C9—H9B	108.9
C3—C2—H2B	111.4	C8—C9—H9B	108.9
C1—C2—H2B	111.4	H9A—C9—H9B	107.7
H2A—C2—H2B	109.2	C14—C10—C1	122.8 (2)
O1—C3—C2	112.74 (18)	C14—C10—C9	120.4 (2)
O1—C3—C4	113.45 (19)	C1—C10—C9	116.8 (2)
C2—C3—C4	103.36 (16)	O4—C11—C12	110.76 (17)
O1—C3—H3	109	O4—C11—C13	105.43 (15)
C2—C3—H3	109	C12—C11—C13	108.42 (17)
C4—C3—H3	109	O4—C11—C7	114.41 (16)
C15—C4—C3	113.75 (17)	C12—C11—C7	101.65 (15)
C15—C4—C5	114.55 (17)	C13—C11—C7	116.10 (17)
C3—C4—C5	103.48 (16)	O3—C12—O2	121.20 (19)
C15—C4—H4	108.3	O3—C12—C11	127.03 (18)
C3—C4—H4	108.3	O2—C12—C11	111.76 (16)
C5—C4—H4	108.3	O5—C13—C11	107.92 (16)
C6—C5—C4	113.89 (15)	O5—C13—H13A	110.1
C6—C5—C1	112.26 (15)	C11—C13—H13A	110.1
C4—C5—C1	105.39 (15)	O5—C13—H13B	110.1
C6—C5—H5	108.4	C11—C13—H13B	110.1
C4—C5—H5	108.4	H13A—C13—H13B	108.4
C1—C5—H5	108.4	C10—C14—H14A	120
O2—C6—C5	108.44 (14)	C10—C14—H14B	120
O2—C6—C7	104.02 (14)	H14A—C14—H14B	120
C5—C6—C7	114.98 (15)	C4—C15—H15B	109.5
O2—C6—H6	109.7	C4—C15—H15A	109.5
C5—C6—H6	109.7	H15B—C15—H15A	109.5
C7—C6—H6	109.7	C4—C15—H15C	109.5
C8—C7—C6	115.08 (17)	H15B—C15—H15C	109.5
C8—C7—C11	116.86 (17)	H15A—C15—H15C	109.5
C6—C7—C11	104.04 (15)		
			
C10—C1—C2—C3	−165.75 (17)	C6—C7—C8—C9	−59.8 (3)
C5—C1—C2—C3	−35.80 (18)	C11—C7—C8—C9	177.73 (18)
C1—C2—C3—O1	170.47 (17)	C7—C8—C9—C10	67.0 (3)
C1—C2—C3—C4	47.57 (19)	C2—C1—C10—C14	−1.2 (3)
O1—C3—C4—C15	72.7 (2)	C5—C1—C10—C14	−124.9 (2)
C2—C3—C4—C15	−164.87 (18)	C2—C1—C10—C9	−179.27 (18)
O1—C3—C4—C5	−162.39 (16)	C5—C1—C10—C9	57.1 (2)
C2—C3—C4—C5	−39.96 (19)	C8—C9—C10—C14	91.2 (3)
C15—C4—C5—C6	−95.3 (2)	C8—C9—C10—C1	−90.7 (2)
C3—C4—C5—C6	140.34 (15)	C8—C7—C11—O4	−86.5 (2)
C15—C4—C5—C1	141.25 (19)	C6—C7—C11—O4	145.49 (17)
C3—C4—C5—C1	16.86 (18)	C8—C7—C11—C12	154.14 (18)
C10—C1—C5—C6	17.1 (2)	C6—C7—C11—C12	26.08 (18)
C2—C1—C5—C6	−112.97 (17)	C8—C7—C11—C13	36.7 (3)
C10—C1—C5—C4	141.60 (18)	C6—C7—C11—C13	−91.3 (2)
C2—C1—C5—C4	11.53 (19)	C6—O2—C12—O3	−179.83 (19)
C12—O2—C6—C5	140.81 (16)	C6—O2—C12—C11	−0.8 (2)
C12—O2—C6—C7	18.0 (2)	O4—C11—C12—O3	40.5 (3)
C4—C5—C6—O2	45.5 (2)	C13—C11—C12—O3	−74.7 (3)
C1—C5—C6—O2	165.20 (15)	C7—C11—C12—O3	162.5 (2)
C4—C5—C6—C7	161.46 (16)	O4—C11—C12—O2	−138.44 (16)
C1—C5—C6—C7	−78.87 (19)	C13—C11—C12—O2	106.34 (18)
O2—C6—C7—C8	−156.38 (16)	C7—C11—C12—O2	−16.5 (2)
C5—C6—C7—C8	85.2 (2)	O4—C11—C13—O5	−170.20 (19)
O2—C6—C7—C11	−27.24 (18)	C12—C11—C13—O5	−51.6 (2)
C5—C6—C7—C11	−145.67 (15)	C7—C11—C13—O5	62.0 (2)

### Hydrogen-bond geometry (Å, °)

**Table:**

*D*—H···*A*	*D*—H	H···*A*	*D*···*A*	*D*—H···*A*
O4—H4O···O1^i^	0.84 (3)	1.93 (3)	2.756 (2)	169 (3)
O5—H5O···O3^ii^	0.86 (3)	1.99 (3)	2.844 (2)	176 (3)
O1—H1O···O3^iii^	0.76 (4)	2.26 (4)	2.978 (3)	159 (4)

Symmetry codes: (i) −*x*, *y*+1/2, −*z*+1/2; (ii) *x*+1/2, −*y*+1/2, −*z*; (iii) −*x*−1/2, −*y*+1, *z*+1/2.
